# Identification of the key exosomal lncRNAs/mRNAs in the serum during distraction osteogenesis

**DOI:** 10.1186/s13018-022-03163-9

**Published:** 2022-05-28

**Authors:** Tao Zhang, Weidong Jiang, Fengchun Liao, Peiqi Zhu, Lina Guo, Zhenchen Zhao, Yan Liu, Xuanping Huang, Nuo Zhou

**Affiliations:** 1 Department of Oral and Maxillofacial Surgery, Hospital of Stomatology, Guangxi Medical University, Nanning, 530021 People’s Republic of China; 2 Guangxi Key Laboratory of Oral and Maxillofacial Rehabilitation and Reconstruction, Guangxi Key Laboratory of Oral and Maxillofacial Surgery Disease Treatment, Guangxi Clinical Research Center for Craniofacial Deformity, Nanning, 530021 People’s Republic of China

**Keywords:** Exosomes, Distraction osteogenesis, JBMMSCs, Osteogenesis, RNA-sequence

## Abstract

**Background:**

Distraction osteogenesis (DO), a kind of bone regenerative process, is not only extremely effective, but the osteogenesis rate is far beyond ordinary bone fracture (BF) healing. Exosomes (Exo) are thought to play a part in bone regeneration and healing as key players in cell-to-cell contact. The object of this work was to determine whether exosomes derived from DO and BF serum could stimulate the Osteogenic Differentiation in these two processes, and if so, which genes could be involved.

**Methods:**

The osteogenesis in DO-gap or BF-gap was evaluated using radiographic analysis and histological analysis. On the 14th postoperative day, DO-Exos and BF-Exos were isolated and cocultured with the jaw of bone marrow mesenchymal stem cells (JBMMSCs). Proliferation, migration and osteogenic differentiation of JBMMSCs were ascertained, after which exosomes RNA-seq was performed to identify the relevant gene.

**Results:**

Radiographic and histological analyses manifested that osteogenesis was remarkably accelerated in DO-gap in comparison with BF-gap. Both of the two types of Exos were taken up by JBMMSCs, and their migration and osteogenic differentiation were also seen to improve. However, the proliferation showed no significant difference. Finally, exosome RNA-seq revealed that the lncRNA MSTRG.532277.1 and the mRNA F-box and leucine-rich repeat protein 14(FBXL14) may play a key role in DO.

**Conclusions:**

Our findings suggest that exosomes from serum exert a critical effect on the rapid osteogenesis in DO. This promoting effect might have relevance with the co-expression of MSTRG.532277.1 and FBXL14. On the whole, these findings provide new insights into bone regeneration, thereby outlining possible therapeutic targets for clinical intervention.

## Introduction

After an intentional surgical bone fracture to preserve the blood supply, distraction osteogenesis is the biological phenomenon involving new bone development among segments of bones that are progressively separated by incremental traction [1]. The DO bone formation rate is extremely fast, in fact, 4 to 8 times that of the adolescent's rapid growth period of epiphysis bone formation [2], and far exceeds the normal rate of fracture healing. In addition, the regional blood flow during DO is 10 times that of the contralateral side [2]. Additionally, the metabolism of DO is more active than fracture healing.

Extracellular vesicles (EVs), or membrane-derived nano- and microvesicles, are secreted by both prokaryotic and eukaryotic cells in an evolutionarily conserved manner [3]. Exosomes are exocrine vesicles having dimensions ranging between 50 and 150 nm [4]. They were first discovered during the study of the red blood cells of sheep in the 1980s. At that time, they were thought to be a way for cells to dispose off garbage [5], but later, a multitude of studies demonstrated that almost all cells secrete EVs into the circulatory system and they possess important intercellular communication functions [6]. The actual function of exosomes depends on the proteins, lipids, and nucleic acids carried by them [7–9].

Exosomes play an essential role in bone formation, which has a close association with the maintenance of homeostasis within the entire body. For instance, the serum of patients with polyarticular juvenile idiopathic arthritis can potentially inhibit the proliferation, differentiation, and mineralization of human osteoblasts (hOb), and promote apoptosis [10]. During the process of distraction osteogenesis, various growth factors in peripheral blood serum, such as MMP-1, TGF-beta1, VEGF, bFGF, hGH, etc., were substantially high in comparison with those before surgery or in comparison with the fracture group at the same time [11–16]. However, there has not been any research to investigate whether or not these factors are released through exosomes. Mari Sato et al. [17] were the first to discover that exosomes secreted by bone cells can enter the blood circulation and perform certain functions. A number of studies have shown that the integration of biomaterials and exosomes has achieved good experimental results in terms of bone defect repair [18–21]. The study of Jia et al. [22] showed that exosomes released by endothelial progenitor cells derived from bone marrow can promote tibial distraction osteogenesis.

As a type of non-hematopoietic stem cell, mesenchymal stem cells (MSC) are originated within the bone marrow. MSCs possess the ability to transform into mature mesenchymal tissue cell kinds such as bone, cartilage, and fat [23]. MSCs were later discovered in all bodily tissues and organs, including peripheral blood, adult and fetal bone marrow, spleen, bone and cartilage, muscle and periodontal tissues, and so forth [24]. A good volume of research has revealed that in comparison with the stem cells coming from other tissues, JBMMSCs have many different characteristics. In humans and rodents, compared with stem cells derived from ilium and tibia, JBMMSCs exhibit stronger proliferation and survival characteristics, and have a significantly stronger osteogenic ability in vivo and in vitro [25, 26]. In rodents, directly implanting mandibular-derived mesenchymal stem cells onto the scaffold can form more new bones than tibia-derived mesenchymal stem cells or a single scaffold graft [26]. Specifically, in distraction osteogenesis, the postoperative complications of distraction osteogenesis of limb bones are more than that of mandibular distraction osteogenesis, and the main complication is non-union [27].

In terms of protein synthesis and transport, chromosome replication, RNA processing and modification, transcriptional control, and other fundamental biological functions, RNA is an essential component of the cellular machinery [28]. Biomedicine has now entered the era of omics, RNA-Seq has been widely used in many studies to clarify the pathophysiological mechanisms in a number of organisms and some related biological processes [29–32]. To date, there does not exist research on the proliferation and differentiation of JBMMSC by serum exosomes after mandibular distraction osteogenesis, and there is no research on serum exosomal RNA in dogs with mandibular distraction osteogenesis.

In our study, exosomes derived from dog serum of mandibular distraction osteogenesis and bone fracture 14 days after surgery (DO-Exos and BF-Exos) were allowed to incubate with the JBMMSCs. The results demonstrated that DO-Exos and BF-Exos did not have any effect on the proliferation of JBMMSCs, but they can promote the migration and osteogenic differentiation of JBMMSCs, and DO-Exos has definitely a stronger effect.

## Materials and methods

### Animal model preparation

The Committee for Animal Care and Use at Guangxi Medical University duly passed all experiments performed on animals. Six stable adult male beagle dogs were split into two categories at random: distraction osteogenesis (DO) and bone fracture (BF). All of the animals were held in a climate-controlled setting (25 °C, steady humidity) within cases made of iron and had free access to regular chow and sterile water.

The canine model was developed in the same way that it had been in previous studies [33, 34]. The dogs in the DO category were anaesthetized with xylazine (2 mg/kg) and pentobarbital (1 mg/kg) intraperitoneally. Following shaving, the site of the operation site was sterilized using iodine (0.5%), and an injection of a mixture comprising lidocaine (0.5%) and 1: 200,000 epinephrine was given. On the right side, an incision of 5-cm was created through the skin from the midline on the inferior borderline of the mandible, and dissection was performed via the subcutaneous and muscular layers. The periosteum was later carefully dissected to reveal the lateral part of the mandible. On the mandibular first and second molars, an osteotomy line was drawn. Following that, the distraction fixator was mounted so that the initial mandibular position could be evaluated after osteotomy. It was necessary for avoiding damage to the inferior alveolar neurovascular bundle during osteotomy. An internal distraction fixator was employed to fix the distal and proximal segments after the completion of the osteotomy (Cibei, China). Finally, the mandibular skin was closed with 4/0 polyglactin absorbable sutures. The animals in the BF community had mandibular osteotomy in an identical place, and the operating area was pulled to 7 mm and fitted with titanium plates after the osteotomy (Cibei, China). After surgery, the dogs were given tramadol hydrochloride and cephalosporin intramuscularly for three days. Distraction was started 7 days after surgery and continued at a 1-mm/day pace in two 12-h measures for 7 days. Six dogs were euthanized for examination on day 14 following surgery.

Blood samples were collected by venipuncture into a blood collection tube without any anticoagulant before DO surgery (DO1 groups), 14 days after DO surgery (DO2 groups), and 14 days after BF surgery (BF2 groups). Blood samples were allowed to sit for 30–60 min at room temperature to fully clot and centrifuged at 4 °C (3000 g, 15 min) for the collection of serum.

### Radiographic analysis

Mandibles were collected, and the attached soft tissue and distractors/titanium plates were removed thoroughly. Micro-computed tomography (micro-CT; Latheta LCT-200, Hitachi Aloka Medical, Nagasaki, Japan) was carried out for measuring the regenerated bone in the distraction/fracture region. Then, bone mineral density (BMD) was assessed by the software of Latheta LCT-200.

### Histology analysis

For 4 weeks, the mandibular specimens were decalcified by employing EDTA (10%, pH 7.0; Solarbio) and embedded in paraffin after being fixed in paraformaldehyde (4%, Solarbio, Beijing, China) for 24 h at room temperature. A rotary microtome was used to segment samples at a thickness of 4-μm. (RM2255, Leica, Germany). With an inverted microscope, these parts were deparaffinized with xylene, hydrated with an ethanol gradient, and stained using H and E or Masson's trichrome (Baso, China) to assess regeneration efficiency. Professional pathologists evaluated H&E and Masson stained sections in a blinded manner.

### Isolation and identification of exosomes

The exosomes were isolated from the DO2 groups and BF2 groups. To reduce the viscosity of the serum, phosphate-buffered saline was used for its dilution and the dilution was performed five times. In order to remove cells and cellular waste, the diluted serum specimens were subjected to centrifugation at 500 × g for 5 min and 2000 × g for 10 min. Centrifugation of the supernatant was carried out at 10,000 × g for another 30 min. The pellet was allowed to resuspend in PBS after being filtered with a 0.22 μm filter and ultracentrifugation (BECKMAN COULTER OptimaTM XE, USA) was performed for 90 min at 110,000 × g. The resuspended solution was later ultracentrifuged for another 90 min at 110,000 × g. After removing the supernatant, resuspension of the final pellet was carried out in PBS 3 mL and it was later concentrated at 4000 × g using the Millipore Amicon Ultra-15 Centrifugal Filter Unit. When the volume reached 100 μL, the process was stopped. Both of the experiments were carried out at 4 °C, and the exosomes were held at − 80 °C.

Nanoparticle tracking analysis (NTA), transmission electron microscopy (TEM), and western blotting were employed for identifying exosomes.

#### Transmission electron microscopy

A transmission electron microscope with negative staining was used to examine the exosome morphology. The exosomal suspension in excess was extracted with great care using a filter paper after 10μL of purified exosomes were loaded onto a grid of copper for 5 min. Uranyl acetate (2%) was used to stain the absorbed exosomes for 1 min, and the excess fluid was filtered out. After the grids had dried, the images of exosomes were captured making use of a transmission electron microscope (TEM, HITACHI H-7650, Japan).

#### Nanoparticle tracking analysis (NTA)

At Viva Cell Biosciences, we used the ZetaView PMX 110 (Particle Metrix, Meerbusch, Germany) and the parallel program ZetaView 8.04.02 to calculate exosome concentration and size of particles by employing nanoparticle tracking analysis (NTA). To test concentration and particle size, isolated exosome samples were diluted with 1X PBS buffer (Biological Industries, Israel). At 11 different locations, NTA measurements were taken and analyzed. Calibration of the ZetaView device was performed using polystyrene particles with a diameter of 110 nm. The temperature was held at about 23 °C.

#### Western blot analysis (WB)

Lysis of the exosomes using EV-specific lysis buffer (Umibio, Shanghai, China) and quantified on a western blot using a BCA protein assay reagent pack (Beyotime). On a 10% sodium dodecyl sulfate–polyacrylamide gel, the protein was isolated and shifted to a 0.22 μm polyvinylidene difluoride (PVDF, Millipore, Billerica, MA, USA) membrane for 1 h at 200 mA. 5% skimmed milk was used to block the membranes for 1 h at room temperature. The blots were probed with primary antibodies overnight at 4 °C, then allowed to incubate with secondary antibodies at room temperature for an hour. The primary antibodies used were CD63 (ABclonal, 1:1000), TSG101 (Signalway Antibody, 1:1000) and Calnexin (Signalway Antibody, 1:1000). After each step, three 10-min washes in 1TBST were performed. Protein bands were then detected in these samples using an ECL reagent (Beyotime).

### JBMMSCs isolation and culture

Mandibular bone marrow and a cancellous bone fragment were used to separate Cannie JBMMSCs. To culture JBMMSCs, we used the whole bone marrow culture process and purified the cells using differential adherent time. α-minimum necessary medium (α-MEM; Gibco, USA), 100 U/mL penicillin, and 100 μg/mL streptomycin, and 10% fetal bovine serum (Gibco, USA) were used to culture the depurated cells. The cells were subjected to incubation at 37 °C and 5% CO_2_ in a humidified atmosphere. The first media exchange washed away non-adherent cells and debris after 72 h. The cell culture medium was modified every 3 days, and the experiments used cells from passages 3–5.

### Multilineage differentiation

#### Adipogenic differentiation

6-well plates were used to culture 3.0 × 10^5^ JBMMSCs (P3) cells with α-MEM without inducers until they reached confluence. α-MEM supplemented with FBS (10%), double antibiotics (1%), indomethacin (0.2 mM), 3isobutyl-1-methylxanthine (0.5 mM), insulin (10 μg/mL), and dexamethasone (1 μM) were used to induce lipid differentiation for 3 days. The cells were cultured for two days with a full medium having 10 μg/mL insulin the next day. The complete process took a duration of 21 days. The JBMMSCs were then painted using Oil Red O after being fixed with 4% paraformaldehyde (Solarbio).

#### Osteogenic differentiation

JBMMSCs (P3) at a density of 1 × 10^4^ cells/cm^2^ were cultured in α-MEM in 6-well or 12-well plates without inducers until 70% confluence. α-MEM medium containing 10% FBS, 1% L-glutamine, 10 mM-phosphoglycerol, 50 μg/mL ascorbic acid, and 10 nM dexamethasone was used to induce osteogenic differentiation for 14 days. Every three days, the induction medium was changed. Following that, the cells were stained with Alizarin Red S. (ARS, 2%, pH 4.2, Beyotime, Shanghai, China) after being fixed in 70% ethanol. Nonspecifically bound and unbound stains were extracted with copious distilled water rinsing, followed by microscopic identification of the stained nodules.

#### Chondrogenic differentiation

Dog Mesenchymal Stem Cell Chondrogenic Differentiation Medium (Cyagen, Guangzhou, China) was used to induce P3 JBMMSCs according to the manufacturer's instructions. 3 × 10^5^ JBMMSCs are used to form one chondrogenic pellet. The pellets were fixed with 4 percent paraformaldehyde overnight after 3 weeks, and parts were painted with Alcian Blue.

### Cytometry

P3 JBMMSCs were digested using trypsin before their resuspension in α-MEM with 10% FBS. Phosphate-buffered saline (PBS) was employed for washing the cells twice. Following this, the cells were incubated for 30 min with antibodies against CD45-eFluor 450, anti-CD34-PE, anti-CD44-FITC, anti-CD146-PE, anti-CD90-APC (Invitrogen, USA), and anti-CD31-FITC (Bioss, China). The cell suspension was then subjected to centrifugation for 5 min at 1000 rpm. Lastly, the cell suspension was moved to a fresh detection tube, and cell surface antigen was detected making use of flow cytometry (BD Biosciences, USA).

### Exosome uptake

JBMMSCs take up DO-exosomes and BF-exosomes. The procedure for isolating exosomes was identical to the one mentioned previously. Exosomes were stained for 10 min with a red fluorescent dye (PKH26, Sigma-Aldrich, USA) and then put through ultracentrifugation at 110,000 × g for 70 min with the supernatant discarded. The exosomes-PKH26 were then incubated with JBMMSCs at 37 °C for 12 h. After that, the cells were washed in PBS and 4 percent paraformaldehyde was used to fix them for 30 min. Following PBS washing (thrice), the cells were stained for three minutes with 4, 6-diamidino-2-phenylindole (DAPI, Solarbio, China). The stained cells were then photographed using a confocal microscope (Leica, Germany).

### CCK-8

At 1, 2, 3, and 4 days, the viability of JBMMSCs was ascertained by employing a Cell Counting Kit-8 (CCK-8) assay (Boster). Using a microplate spectrophotometer, the count of viable cells was made by estimating the optical density (OD) value at 450 nm in 5 wells per community (Infinite MFlex, TECAN).’

#### Transwell

To measure cellular migration, researchers used Transwell assay inserts (Corning, NY, USA). In a 24-well plate, JBMMSCs (8 × 10^3^ cells in 400 μL serum-free media) were attached to the upper part of the inserts, with 750 μL of media containing various exosomes at a concentration of 50 μg/ml in the bottom chamber. Cells were allowed to culture for 36 h, during which all cells that had migrated to the bottom chamber were fixed with 4% neutral-buffered formalin, followed by staining using crystal violet (0.1%, Solarbio). The cells were then counted and imaged using a microscope.

#### Alkaline phosphatase(ALP) staining and ARS staining

The three categories of JBMMSCs were plated into 6-well or 12-well plates at a density of 1 × 10^4^ cells/cm^2^ and cultured until the cells reached 70% to 80% confluence. For the next three days, cells were subjected to pretreatment with a mineralization medium containing various treatments (control group, DO-Exo group, and BF-Exo group). The concentration of exosomes is 50 μg/ml. Finally, the cells were cultured with a mineralization medium after being pretreated. After that, the cells were immobilized, and ALP activity was measured after 8 days while employing an ALP staining kit (Beyotime, Shanghai, China). At 14 days, alizarin red S (Beyotime, Shanghai, China) staining was used to assess bone mineralization. Finally, hexadecyl pyridinium chloride (Sigma) was used to dissolve the mineralized nodules, and absorbance was determined quantitatively at 560 nm for statistical analysis.

#### Serum-exosomes RNA-seq

Before DO surgery (DO1 groups), 14 days after DO surgery (DO2 groups), and 14 days after BF surgery (BF2 groups), serum was collected. Total RNA from serum-exosomes was extracted in light of the instructions provided by the manufacturer by making use of a Trizol reagent kit (Invitrogen, Carlsbad, CA, USA). RNase-free agarose gel electrophoresis was used to verify RNA content using an Agilent 2100 Bioanalyzer (Agilent Technologies, Palo Alto, CA, USA). After extracting complete RNA, rRNAs were removed to leave only mRNAs and ncRNAs. Using fragmentation buffer, the enriched mRNAs and ncRNAs were broken down into smaller fragments and reverse transcribed into cDNA using random primers. DNA polymerase I, dNTP (dUTP instead of dTTP), RNase H, and buffer were utilized to make second-strand cDNA. The cDNA fragments were then purified using a QiaQuick PCR extraction kit (Qiagen, Venlo, The Netherlands), end-repaired, and base attached, and ligated to Illumina sequencing adapters. After that, the digestion of the second-strand cDNA was carried out while employing UNG (Uracil-N-Glycosylase). Gene Denovo Biotechnology Co. agarose gel electrophoresis was used to size pick the digested products and then PCR amplified and sequenced them by making use of the Illumina Novaseq6000 (or other platforms) (Guangzhou, China). Fastp (version 0.18.0) was employed for filtering the raw data for downstream analysis. By default parameters, the protein-coding ability of novel transcripts was evaluated using two software programs: CNCI (version 2) and CPC (version 0.9-r2) (http://cpc.cbi.pku.edu.cn/). Long non-coding RNAs were chosen as the intersection of both non-protein-coding possible outcomes. The DESeq2 program was used to compare the differential expression of RNAs and lncRNAs between the two types. P-value 0.05 and fold change 2 were used to describe significantly different expressions. DEGs were analyzed using Gene Ontology (GO) enrichment, Kyoto Encyclopedia of Genes and Genomes (KEGG) pathway enrichment, and lncRNA-mRNA interaction analysis. Gene Denovo Biotechnology Co. carried out both of the above procedures (Guangzhou, China).

#### Statistical analysis

The mean and standard deviation are used to represent all values. Statistical studies were carried out using the SPSS 23.0 program. Individual experiments were carried out at least three times. As required, the Student's t-test and one-way ANOVAs were used. Statistical significance was described as a P-value < 0.05.

## Results

### Animal model

**Fig. 1:**
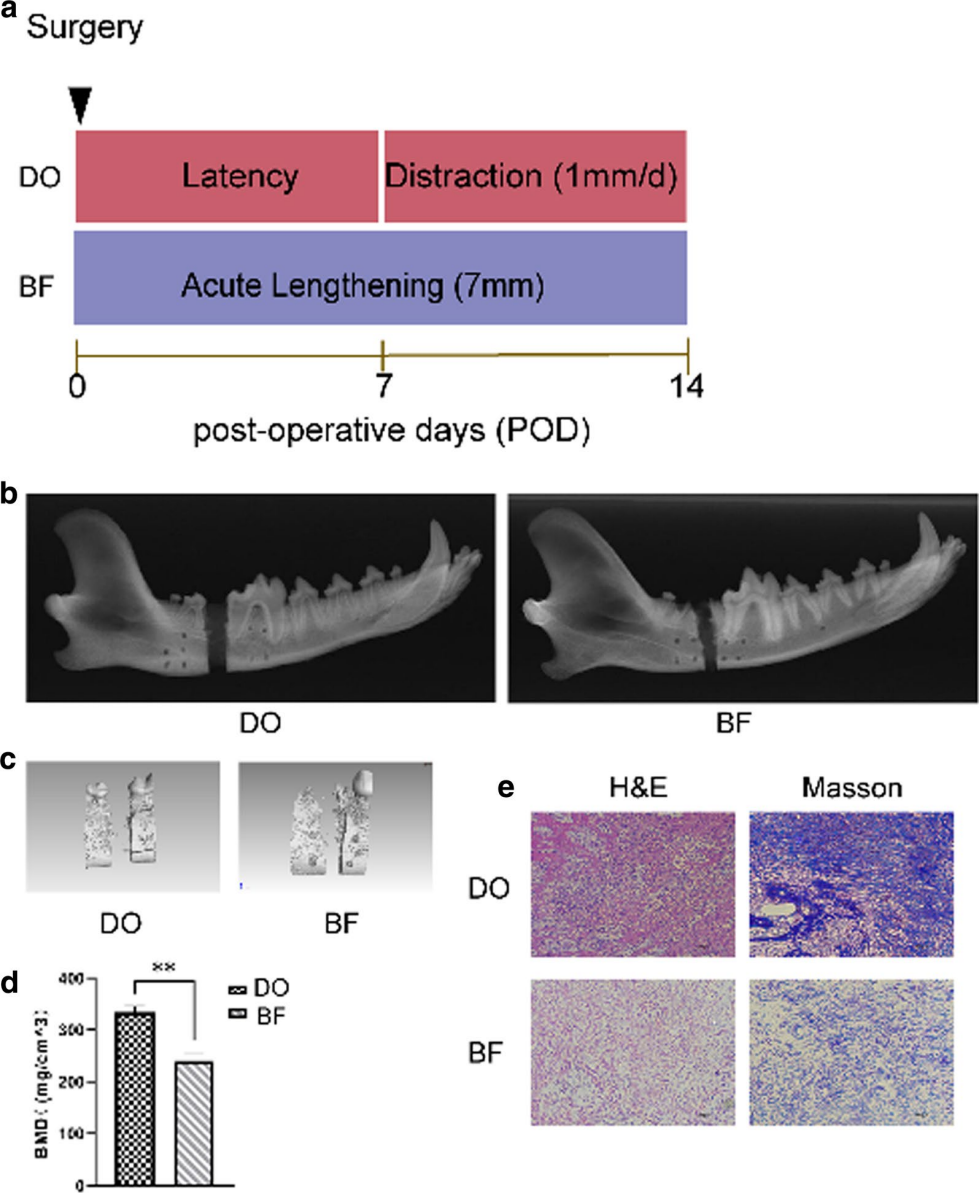
DO has a greater bone formation compared with BF. **a** Schematic diagram of the DO and BF models. **b** Radiographic imaging by X-ray was carried out 14 days after the surgery. **c** Representative micro-CT images of DO and BF gaps. **d** BMD on postoperative day 14 was estimated, *n* = 3. **e** H and E, and Masson staining of the new tissues in the DO and BF gaps. BMD, bone mineral density. **P < 0.01

All operations went well, and the animals survived, so there was no risk of infection (see Fig. 1a).

### Radiographic analysis

The gaps were obvious in both of the groups. But the edges of the gaps in DO groups are rougher than those in BF groups. This indicates that the new bone formation takes place earlier in DO groups than BF groups (see Fig. 1b). For statistical analysis and observation, a region of interest (ROI) in the distraction or fracture gap was chosen for micro-CT analysis. Quantitative analysis revealed that the DO group had significantly higher BMD than the BF group (P < 0.01, see Fig. 1c, d).

### Histological analysis

The new tissues in the DO group included a substantial number of dense fibrous tissues, the orientation of which corroborated with the direction of distraction powers, and the fibrous tissue space manifested large numbers of red blood cells, which was in good agreement with the histological manifestations in the initial stages of angiogenesis. The BF group's new tissue was disordered and fragmented, with a large number of inflammatory cells infiltrating it (see Fig. 1e).

The collagen structure and density were assessed using Masson trichrome staining. The density of collagen fibers in the regenerated tissue of DO groups was higher than that of BF groups (see Fig. 1e). Collagen fibers in DO groups (see Fig. 1e) were arranged along the distraction path and were incorporated with numerous blood vessels. In the BF groups, however, a significant amount of disorderly organized collagen deposits diffused in the gaps were discovered (see Fig. 1e).

When comparing the DO canine to the BF group, histological examination with H&E and Masson staining showed a visible improvement in bone regeneration (see Fig. 1e).

### Characterization of exosomes

**Fig. 2:**
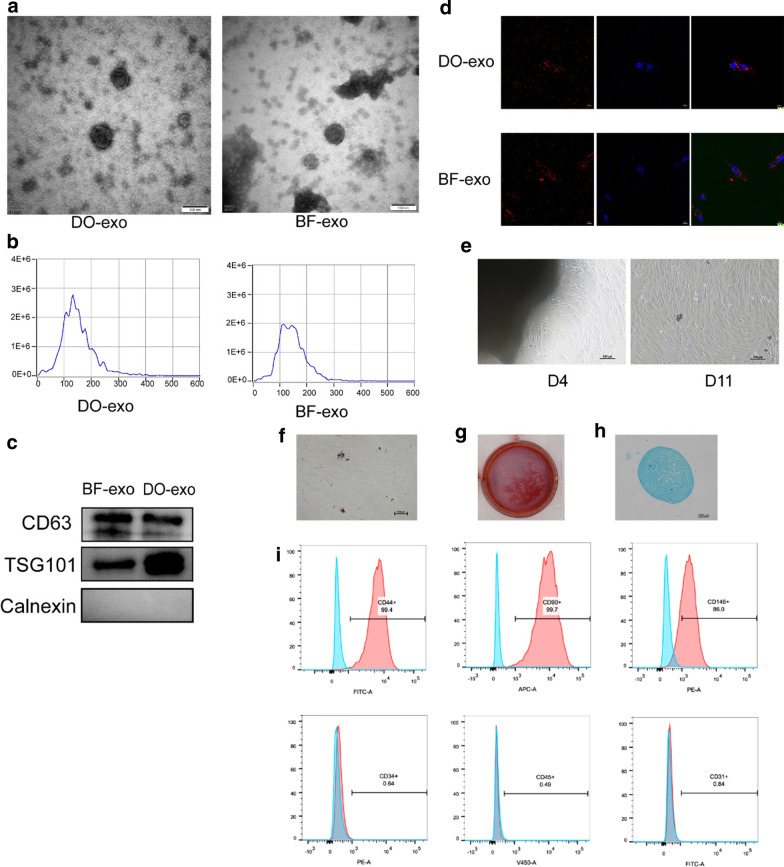
Characterization of DO-Exos, BF-Exos, and JBMMSCs. **a** The morphology of Exos revealed by TEM. Left: morphology of DO-Exos; right: morphology of BF-Exos; **b** the particle size distribution in Exos ascertained by NTA. Left: The particle size distribution in DO-Exo; right: the particle size distribution in BF-Exo; **c** Western blotting was carried out to detect exosome surface markers (CD63 and TSG101). **d** Internalization of PKH26-labeled DO-Exos and BF-Exos by JBMMSCs using laser scanning confocal microscopy. **e** Fusiform morphology of JBMMSCs depicted as light microscopy images. **f** Oil red staining was used to determine the capability of JBMMSCs to differentiate into lipids. **g** The capacity of JBMMSCs to differentiate into osteoblasts was determined using Alizarin red staining. **h** JBMMSCs were stained with Alcian Blue to see whether they could differentiate from cartilage. (i) Flow cytometry was used to examine the surface markers of JBMMSCs. CD90, CD44, and CD146 were all positive, but CD45, CD34, and CD31 were all negative

TEM, NanoSight, and western blotting were used to examine the extracted exosomes. The majority of the particles in both the DO and BF classes had a cup- or round-shaped morphology, according to TEM photos (see Fig. 2a). DO-exosomes had a diameter of approximately 135.5 ± 4.29 nm, while BF-exosomes had a diameter of approximately 121.93 ± 4.44 nm (see Fig. 2b). CD63 and TSG101 proteins were found to be expressed and Calnexin protein was not found to be expressed. (see Fig. 2c). The findings showed that both groups' extracted exosomes had properties that were consistent with generally accepted criteria.

The PKH26 assay was used to confirm exosomes' ability to fuse with cells. After staining, the DO-Exos and BF-Exos were applied to JBMMSCs separately. The stained exosomes treated cells showed PKH26 cytoplasmic fluorescence, according to laser scanning confocal microscopy study (see Fig. 2d). This discovery showed that both groups' purified exosomes could be internalized.

### Characterization of JBMMSCs

The JBMMSCs derived from the canine mandibular had a vortex distribution and a fusiform shape, which is characteristic of fibroblasts with a spindle-shaped morphology (see Fig. 2e). Adipogenic, osteogenic, and chondrogenic differentiation was induced by seeding third passage cells into 6-well plates. Oil red staining revealed a significant number of lipid droplets after 21 days of induction (see Fig. 2f). The findings of the alizarin red staining revealed that there were several calcified nodules (see Fig. 2g). Similarly, the pellets' Alcian Blue staining was positive (see Fig. 2h). Both types of cells were positive for CD44, CD90, and CD146, but negative for CD31, CD34, and CD45, as revealed by flow cytometry data (see Fig. 2i).

### Proliferation of JBMMSCs

**Fig. 3:**
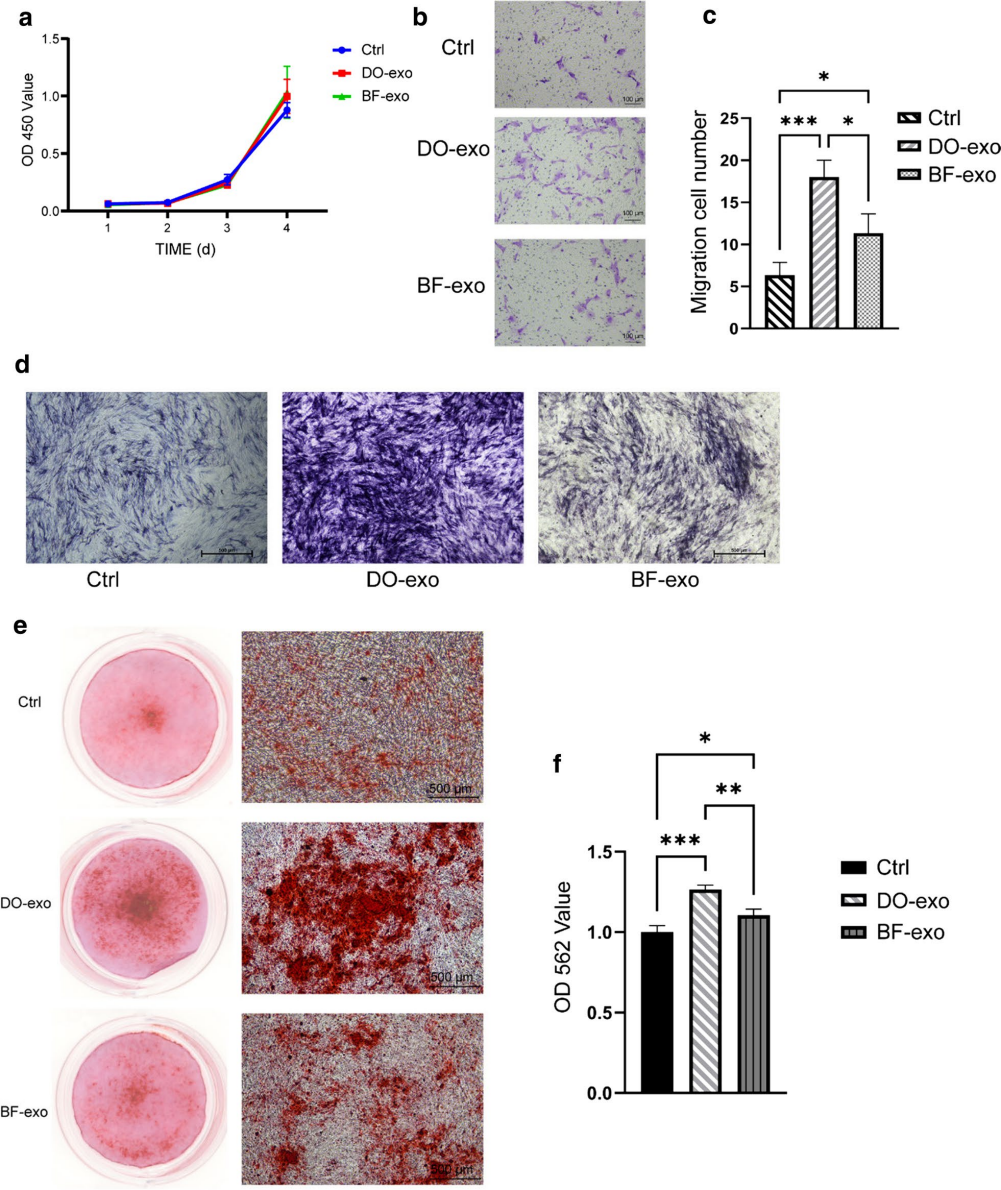
The DO-Exos promote the migration and osteogenic ability of JBMMSCs. **a** CCK-8 assay results revealed that DO-Exos and BF-Exos had no affection on the viability of JBMMSCs. **b**, **c**: Transwell assays showed DO-Exos enhanced migration of JBMMSCs compared with BF-Exos and control groups. **d** images of ALP staining of JBMMSCs, ALP staining at 8 days. **e** Gross, and microscope scanning images of ARS staining at 14 days. **f** Quantification of ARS staining

The CCK-8 assay showed that DO-exosomes and BF-exosomes did not affect JBMMSCs cell growth (see Fig. 3a). (P > 0.05).

### Migration and osteogenesis differentiation of JBMMSCs in vitro

Transwell assays were used to see how DO-exosomes and BF-exosomes affected the ability of JBMMSCs to migrate. The results of transwell assays revealed that both DO-Exos and BF-Exos would substantially improve the migration ability of JBMMSCs, with DO-Exos having a better promotion ability than BF-Exos (see Fig. 3b, c). The ability of JBMMSCs to form a mineralized matrix was assessed using ALP and Alizarin Red S staining. At 8 days, cells treated with DO-exosomes and BF-exosomes had more intense ALP staining than the control group, as shown in Fig. 3d, and DO-exosomes had a stronger promotion potential. At 14 days, alizarin red S staining revealed that the mineral deposition of the DO and BF groups was significantly higher than the control group, and DO-Exos had the greater osteogenic potential of JBMMSCs than BF-Exos (see Fig. 3e, f).

### Transcriptome analysis identified signature gene network in distraction osteogenesis

**Fig. 4:**
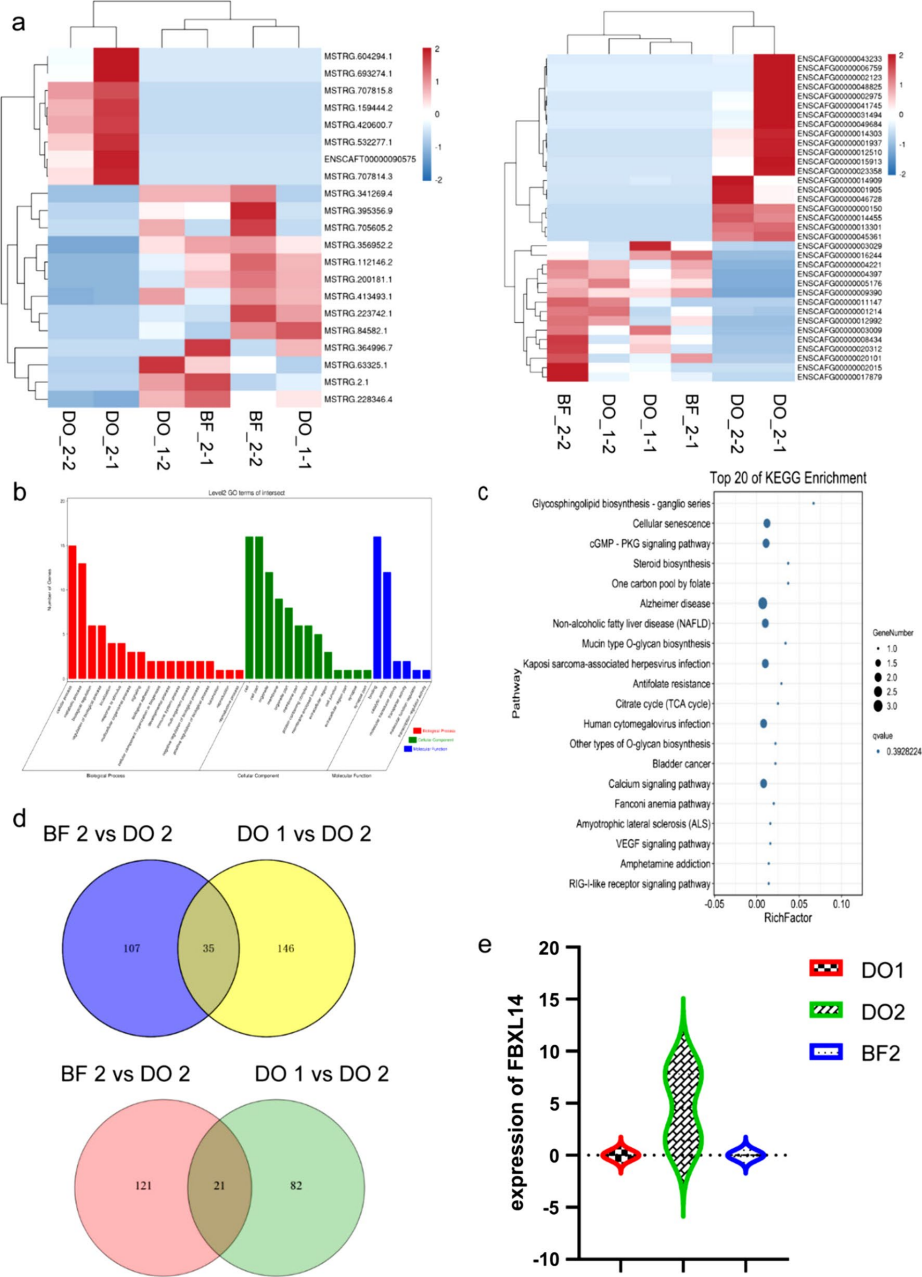
Transcriptome analysis identified the enriched genes in distraction osteogenesis. **a** Heat map of the differentially expressed genes in DO and BF sera exosomes. Exosomes from DO1, DO2, and BF2 sera were analyzed using RNA sequencing (RNA-seq). In these populations, major differences in gene expression were discovered. Cut-off values: P-value < 0.05 and fold change > 2. **b** GO analysis indicated significant enrichment of cellular process, response to stimulus, the developmental process associated genes in DO serum exosomes. **c** Signaling pathway analysis indicated that Cellular senescence, cGMP–PKG signaling path-way, calcium signaling pathway, and VEGF signaling pathways were considerably enriched in DO serum exosomes. **d** The Venn diagram of the differential RNAs of DO2-versus-DO1 and DO2-versus-BF2. **e** Sequence results revealed that FBXL14 increased in expression levels among the three groups

Serum-exosomes RNA-seq was performed in three groups to examine the possible molecular mechanism that leads to functional differences between DO and BF (before surgery, 14 days after DO surgery, and 14 days after BF surgery). Long non-coding RNAs (long ncRNAs, lncRNAs) and differentially expressed mRNAs were discovered. Figures 4a shows the expression heat maps of the differential RNAs, which were created to show the distribution in each sub-class.

GO enrichment analysis and KEGG pathway enrichment analysis were used to obtain a basic understanding of biological functions. The GO enrichment analysis findings revealed that cellular process, response to stimulus, developmental process, etc., were significantly enriched in critical biological functions (see Fig. 4b). The genes are significantly involved in several pathways that are associated with Cellular senescence, Cgmp—PKG signaling pathway, Calcium signaling pathway, and VEGF signaling pathway, as revealed by the KEGG enrichment research (see Fig. 4c). Then, using overlapping analysis, a Venn diagram was created to find overlapping genes among the DO4-vs-DO1 and DO4-vs-BF4 differential RNAs. There were 35 mRNAs and 21 lncRNAs present, as seen in the Venn diagram (see Fig. 4d). To identify the relevant lncRNA and mRNA, co-expression analysis was performed. Therefore, only the lncRNA MSTRG.532277.1 and mRNA FBXL14 were chosen as candidates. mRNA FBXL14 is associated with osteogenesis [35]. And the results of gene expression were consistent with other study (see Fig. 4e). In conclusion, we were able to identify genes that were explicitly expressed in the DO rapid osteogenesis phase using transcriptome analysis.

## Discussion

Distraction osteogenesis is a controllable way of the formation of bone [36] and regardless of whether it occurs within the appendicular or craniofacial skeleton, it resembles intramembranous ossification [1]. In addition, our experiments, in vivo, were proved via imaging and histological analysis. that the bone formation rate of DO is faster than BF. This suggests that distraction osteogenesis and fracture healing are being contrasted as two distinct mechanisms of bone recovery and regeneration [36, 37]. The discovery of the biological mechanism behind it is indeed of great significance, for the repair of bone defects caused by various clinical reasons. Exosomes are extracellular vesicles with a diameter of 50-150 nm, which can carry and transport specific intracellular substances (such as protein lipids and nucleic acids) for inter-cell communication with neighboring or distal cells, and then perform their regulatory functions [38–40]. Exosomes contain transcripts such as mRNA lncRNA miRNAs, known as exosomal RNAs, which have been demonstrated to have important functions in carrying out transfer between different cell types [41–43]. Exosomal RNA has been shown to avoid degradation by intracellular RNA enzymes, and is relatively stable when subjected to temperature changes or even stored for a long time [44, 45]. A growing volume of evidence shows that exosomes have a key involvement in the process of osteogenesis, for example, the exosomes of mesenchymal stem cells act on other mesenchymal stem cells or direct domain osteoblasts in order to promote bone formation [18, 19, 46–48]. But there has been no study about the difference in serum exosomes following fracture and distraction osteogenesis.

We attempted to probe into the systemic influence of serum exosomes collected from DO and BF 14 days after surgery on JBMMSCs proliferation and differentiation in vitro. Osteogenic differentiation was found to increase significantly for both DO-Exos groups and BF-Exos groups, whereas DO serum exosomes were observed to have a stronger ability to promote osteogenic differentiation. JBMMSCs proliferation was not significantly different between DO-Exos and BF-Exos. According to the previous study, MMP-1, TGF-beta1, VEGF, bFGF, hGH, etc., were significantly higher in the serum than those before surgery or in comparison with the fracture group at the same time [11–16]. Quite a lot of evidence supports that MMP-1, TGF-beta1, VEGF, bFGF, hGH can promote the proliferation of MSC [49–52]. However, in our study, JBMMSCs did not manifest enhanced proliferation with DO serum exosomes, suggesting that other circulating factors in serum exosomes may hinder in vitro cell growth. Different from the proliferation of JBMMSCs, DO-Exo can significantly promote the migration and osteogenic differentiation of JBMMSCs. Insights into this phenomenon might have valuable significance for our research on the rapid osteogenesis of DO. Although there has been the RNA-seq of the tissue in the DO and BF gap, osteogenesis is a process involving the system environment; hence, serum-exo RNA-seq is essential for discovering the mechanism of fast bone formation in DO.

The RNA-seq shows that lncRNA MSTRG.532277.1 and mRNA F-box and leucine-rich repeat protein 14 (FBXL14) may play important roles in DO. MSTRG.532277.1 is a predicted long non-coding RNA and can co-express with FBXL14. FBXL14 is one of 21 members of the FBXL subfamily of F-box proteins, all of which have Leucine-rich repeats (LRR) at their C-termini (5 LRRs in the case of FBXL14) [53]. The substrate recognition components of the Skp1-Cul1-F-box-protein (SCF) ubiquitin ligase, the F-box family of proteins (FBPs), are evolutionarily conserved in various organisms and have fundamental involvement in a variety of biological processes, for instance, cell cycle control and growth [54–56]. It was discovered that FBXL14 is expressed in the initiation phase of bone generation in cultured bovine-periosteum-derived cells [35, 57]. Fbxl14 has also been shown to control Xenopus laevis embryonic neural crest production and zebrafish embryonic axis formation [56, 58]. It is well known that mandibular is origin from the neural crest and thus suggests its valuable participation in mandibular regeneration. In our result, FBXL14 was found to have a high expression in DO groups after surgery, in comparison with the BF groups as well as, prior to surgery. The observation corroborates well with the previous reports in the literature. So, MSTRG.532277.1-FBXL14 co-expression may contribute to the rapid osteogenesis in DO. However, further study should be made in order to clarify its detailed signal transduction pathway.

## Conclusions

In conclusion, the bone consistency of DO mandibular regeneration is superior to that of BF gap healing, according to our findings. DO-exosomes could speed up JBMMSC migration and osteogenic differentiation as an intercellular communicator, and MSTRG.532277.1-FBXL14 co-expression could be one of the underlying mechanisms in the distraction osteogenesis phase. The findings are the first to show that canine mandibular distraction osteogenesis and bone fracture cause RNA changes in serum exosomes. Our results indicate that MSTRG.532277.1, FBXL14 may be a prospective therapeutic target in view of rapid bone regeneration, pointing to important clinical research directions.

## Data Availability

The transcriptome data were deposited on NCBI SRA (accession: PRJNA749904), https://dataview.ncbi.nlm.nih.gov/object/PRJNA749904?reviewer=q9sdb7nkf8o36jer754mv5v9hh. The other data are included in this published article.

## Abbreviations

DO: Distraction osteogenesis
BF: Bone fracture
JBMMSCs: Jaw of bone marrow mesenchymal stem cells
FBXL14: F-box and leucine-rich repeat protein 14
EVs: Extracellular vesicles
MSC: Mesenchymal stem cells
Micro-CT: Micro-computed tomography
NTA: Nanoparticle tracking analysis
TEM: Transmission electron microscopy
ARS: Alizarin red S
CCK-8: Cell counting kit-8
ALP: Alkaline phosphatase
